# Informant questionnaire on cognitive decline in the elderly (IQCODE) for assessing the severity of dementia in patients with Alzheimer’s disease

**DOI:** 10.1186/s12877-018-0837-9

**Published:** 2018-06-19

**Authors:** Yunlong Ding, Jiali Niu, Yanrong Zhang, Wenpeng Liu, Yan Zhou, Can Wei, Yan Liu

**Affiliations:** 1 Department of Neurology, JingJiang People’s Hospital, No. 28, Zhongzhou Road, Jingjiang, CN 214500 Jiangsu China; 2 Department of Clinical Pharmacy, JingJiang People’s Hospital, No. 28, Zhongzhou Road, Jingjiang, CN 214500 Jiangsu China; 3 Department of Neurology, Huashan Hospital at Fudan University, No. 12, Urumqi Road, Shanghai, 200040 CN China

**Keywords:** IQCODE, Alzheimer’s disease, Dementia

## Abstract

**Background:**

The Informant Questionnaire on Cognitive Decline in the Elderly (IQCODE) is widely used as a complementary screening tool for dementia. However, there are few studies concerning the efficacy of the IQCODE for assessing the severity of cognitive impairments in patients with Alzheimer’s disease (AD). We aimed to evaluate the efficacy of the IQCODE for assessing the severity of dementia in patients with AD.

**Methods:**

According to the clinical dementia rating (CDR), 394 patients with AD were enrolled and classified into three groups: mild, moderate and severe groups. The IQCODE scores of each group were determined by interviewing the informants with the short version of the 16-item IQCODE. The correlations of the IQCODE score with the Mini-Mental State Examination (MMSE), the Mattis Dementia Rating Scale (DRS) and the Alzheimer’s Disease Assessment Scale-Cognitive Subscale (ADAS-Cog) were analysed. Statistical analyses were conducted to examine the differences in the IQCODE scores among the three groups.

**Results:**

The validity coefficients of the IQCODE with the MMSE, DRS and ADAS-Cog were − 0.528, − 0.436, and 0.477, respectively. The sensitivity was 66.1%, and the specificity was 59.8% when using a cut-off score of 65 to discriminate between mild-moderate dementia. When 75 was used as the threshold between moderate-severe dementia, the sensitivity and the specificity were 73.9 and 67.7%, respectively.

**Conclusions:**

The IQCODE is moderately effective for assessing the severity of cognitive impairment in patients with AD.

## Background

Alzheimer’s disease (AD) is the most common form of neurodegenerative disease associated with dementia among the elderly [1]. AD is characterized by a progressive decline in cognitive function that often begins with early disturbances in episodic memory and ultimately leads to absolute functional impairment [2, 3]. Currently, the severity of cognitive dysfunction in patients with AD is often assessed with various instruments, including the Clinical Dementia Rating (CDR), the Mini-Mental State Examination (MMSE) and patient-administered assessment tools in clinical trials and clinical studies [4]. Unfortunately, the effectiveness of these assessments may be influenced by several factors such as age, gender, educational level, and premorbid intelligence of the patients [5–8].

The Informant Questionnaire on Cognitive Decline in the Elderly (IQCODE) uses collateral information to assess changes in everyday cognitive functions over the previous 10-year period [9]. The IQCODE is based on a structured interview in which responses of informants who are often the spouses or relatives of patients who know the patients well are collected [10]. As an informant questionnaire, the IQCODE asks a series of questions about how the patient’s cognition and functioning have changed, and as a result, it is not affected by the patient’s premorbid intelligence or education [8]. Currently, several studies have already shown that the IQCODE is widely effective as a complementary screening test for dementia and is used within various cultural contexts, including Australian, Spanish, Lebanese, and Chinese [9, 11–17]. In a study of a Singaporean population, for instance, the combination of cognitive testing and the IQCODE provided better detection of dementia when individuals had no education [18]. Additionally, a study using a Spanish sample found that the IQCODE was more sensitive for identifying mild dementia than the MMSE [17]. A short form of the IQCODE that contained 16 items was subsequently developed; this version was highly correlated with the original version and possessed comparable validity [16]. The IQCODE is widely used as a complementary screening tool for dementia. However, there are few studies that have evaluated the efficacy of the IQCODE for assessing the severity of cognitive impairments in patients with AD.

Therefore, this study recruited AD patients and interviewed their informants with the 16-item IQCODE to explore its clinical value for assessing the severity of cognitive impairments in patients with AD.

## Methods

### Study participants

A total of 394 participants who were admitted to Jingjiang People’s Hospital (201 cases) and Huashan Hospital at Fudan University (193 cases) from January 2013 to August 2016 were included in the study. Among all the patients, there were 184 males and 210 females. The patients’ ages ranged from 43 to 91 years, with a mean age of 70.01 years (SD = 9.77), and their education levels varied from illiterate to bachelor’s degrees. Following a series of examinations, including a medical history, a clinical examination, neuropsychological testing and laboratory assessments, all the patients’ conditions met the criteria for “the diagnosis of dementia due to Alzheimer’s disease” that was recommended by the National Institute on Ageing-Alzheimer’s Association workgroups [19]. All the informants, including 128 spouses, 215 children and 51 other relatives, were individuals who had been living with the patients for more than 10 years, contacted them at least 4 times a week and knew the patients’ information well. Informed consent was obtained from participants with the capacity to consent. If they did not have capacity, a guardian or close family member was asked to give agreement for the person’s participation, and sign his/her agreement for this. The experiment was approved by the local ethics committee.

### Procedure

Three hundred and ninety-four patients with AD were classified into the following three groups by two senior neurologic physicians according to the severity of dementia as assessed by the CDR scores [20]: mild (CDR = 1.0, *n* = 107), moderate (CDR = 2.0, *n* = 194) and severe (CDR = 3.0, *n* = 93) dementia groups. If the two physicians had different opinions on the grouping, a third senior neurologic physician intervened until an agreement was reached. Each patient was assessed by a geriatrician who administered the MMSE [21], the Mattis Dementia Rating Scale (DRS) [22], and the Alzheimer’s Disease Assessment Scale-Cognitive Subscale (ADAS-Cog) [23]. In addition, the informants were interviewed with the short version of the IQCODE containing 16 items.

Cognitive functions that were tested with the short IQCODE included short- and long-term memory, space and time orientation, calculation, learning and administration. Each item was ranked on a 5-point scale with 1 representing “much improved”, 2 representing “a bit improved”, 3 representing “not much change”, 4 representing “a bit worse” and 5 representing “much worse”. The sum of the scores was averaged over the 16 items to yield a total score ranging from 1 to 5, with higher scores representing greater impairment [16].

### Statistical analysis

Statistical analysis was performed with SPSS version 17.0 software (SPSS, Chicago, IL, USA). The data are presented as the mean ± standard deviation (SD). Agreement between the 2 independent physicians was determined using kappa statistics, and κ values ranging from 0.8 to 1.0 and from 0.6 to 0.8 were considered to represent nearly perfect agreement and substantial agreement, respectively. Clinical characteristics were evaluated using χ^2^ tests. Statistical differences between subject groups were tested using Student’s t-test for normally distributed variables or Fisher’s exact test for dichotomous variables. A receiver operating characteristic (ROC) curve analysis was employed to compare the accuracy of the IQCODE for assessing the severity of cognitive impairment in patients with AD. The area under the curve (AUC) was used as a measure of the dichotomous screening ability of the test and for choosing an optimal cut-off point that provided the largest AUC. The clinical significance was evaluated by analysing the correlation of the IQCODE scores with the MMSE scores, the DRS scores and the ADAS-Cog scores. Values of *P* less than 0.05 were considered statistically significant.

## Results

### Inter-observer variability

The agreement of two senior neurologic physicians’ in judging the severity of dementia was excellent, with a κ value 0.894 (*P* < 0.001). The third senior neurologic physician intervened when they disagreed on the group until an agreement was reached.

### Clinical characteristics

The participant characteristics are summarized by each stage of dementia in Table 1. A total of 394 AD patients participated in this study, of which 107 (27.2%) patients were in the mild group, 194 (49.2%) patients were in the moderate group, and 93 (23.6%) patients were in the severe group. No significant differences were observed owing to age and sex among these three groups. However, the education levels in the mild, moderate and severe dementia groups were 6.21 (±1.70), 5.60 (±1.87), and 5.39 (±1.99), respectively, indicating that education level was negatively correlated with the severity of dementia (*P* = 0.004). The differences among the MMSE, DRS, and ADAS-Cog scores were statistically significant.

**Table 1:** General materials of 394 cases (mean ± sd)

items	Mild dementia (*n* = 107)	Moderate dementia (*n* = 194)	Severe dementia (n = 93)	F (*P*)
Age in years	70.10 ± 9.22	70.57 ± 10.05	68.72 ± 9.77	1.137(0.322)
sex (male:female)	52:55	90:104	42:51	0.124(0.883)
Educationg level	6.21 ± 1.70	5.60 ± 1.87	5.39 ± 1.99	5.506(0.004)
MMSE	19.62 ± 2.04	14.86 ± 2.96	8.29 ± 3.25	402.806(0.000)
DRS	112.75 ± 15.25	88.80 ± 13.16	67.52 ± 15.86	236.362(0.000)
ADAS-cog	29.10 ± 11.08	40.45 ± 12.17	53.27 ± 9.38	89.686(0.000)

Education level: 1.Illiteracy, 2. Old-style private school or family education, 3.Adult literacy class or night school, 4. Primary school drop-outs, 5.Primary school, 6.junior high school, 7.senior high school, 8.College or above

*Abbreviations*: *MMSE* the mini-mental state examination, *DRS* the Mattis dementia rating scale, *ADAS-cog* the Alzheimer’s disease assessment scale-cognitive subscale

### Correlation between the IQCODE scores and neuropsychological testing

Our results showed that the correlation coefficients of the IQCODE scores of the mild and severe dementia groups with neuropsychological testing, including the MMSE, DRS, and ADAS-Cog scores, were not statistically significant. However, the correlation coefficients of the IQCODE scores of moderate dementia with these three neuropsychological tests, the MMSE, DRS, and ADAS-Cog, were statistically significantly different (− 0.409, − 0.324, and 0.370, respectively). Furthermore, significant differences were observed in the total sample, and the correlation coefficients with the MMSE, DRS, and ADAS-Cog were − 0.528, − 0.436, and 0.477, respectively (Table 2).

**Table 2:** Correlation of IQCODE scores and neuropsychological testing

	MMSE	DRS	ADAS-cog
Mild dementia	0.009	0.115	0.137
Moderate dementia	−0.409^**^	− 0.324^**^	0.370^**^
Sever dementia	−0.131	−0.193	0.197
All	−0.528^**^	−0.436^**^	0.477^**^

*Abbreviations*: *MMSE* the mini-mental state examination, *DRS* the Mattis =dementia rating scale, *ADAS-cog* the Alzheimer’s disease assessment scale-cognitive subscale

^*^P < 0.05, ^**^P < 0.01

### Effectiveness of the IQCODE in assessing the severity of cognitive impairment

In the primary analysis, dementia of different severities had significantly different IQCODE scores for items, except the third item. The total scores from the mild, moderate and severe groups were 63.38 (±9.49), 68.39 (±8.73) and 75.96 (±3.52), respectively, and were statistically significant (Table 3). For discriminating between mild-moderate dementia, the ROC curve analyses for the IQCODE showed an AUC of 0.666 (95% confidence interval (CI), 0.601–0.732). With 65 used as the cut-off point, the sensitivity was 66.1%, and the specificity was 59.8%. The AUC under the ROC curve for the IQCODE discriminating between moderate and severe dementia was 0.768 (95% CI, 0.715–0.822); the sensitivity was 73.9%, and the specificity was 67.7% with 75 used as the cut-off score.

**Table 3:** Comparison of single item and total IQCODE score of different severity of dementia

iterm	Mild dementia (*n* =107)	Moderate dementia (*n* = 194)	Severe dementia (*n* = 93)	F (*P*)
1	4.07 ± 0.72	4.32 ± 0.76^**^	4.92 ± 0.30^**^	43.820(0.000)
2	4.58 ± 0.80	4.79 ± 0.56^*^	4.94 ± 0.25^**^	9.474(0.000)
3	4.73 ± 0.56	4.81 ± 0.52	4.92 ± 0.27^*^	3.763(0.024)
4	3.71 ± 1.01	4.34 ± 0.92^**^	4.71 ± 0.72^**^	32.130(0.000)
5	3.60 ± 0.93	3.51 ± 0.79	4.00 ± 0.88^**^	10.832(0.000)
6	3.74 ± 0.92	4.07 ± 0.79^**^	4.59 ± 0.61^**^	29.307(0.000)
7	3.92 ± 0.84	4.25 ± 0.77^**^	4.80 ± 0.48^**^	36.338(0.000)
8	3.75 ± 0.89	4.11 ± 0.81^**^	4.70 ± 0.62^**^	36.349(0.000)
9	3.86 ± 0.92	4.27 ± 0.72^**^	4.77 ± 0.53^**^	37.726(0.000)
10	3.94 ± 0.88	4.34 ± 0.72^**^	4.86 ± 0.46^**^	40.532(0.000)
11	4.59 ± 0.75	4.78 ± 0.60^*^	4.91 ± 0.32^*^	7.711(0.001)
12	3.62 ± 0.78	3.86 ± 0.91^*^	4.51 ± 0.62^**^	31.684(0.000)
13	3.71 ± 0.88	4.08 ± 0.91^**^	4.75 ± 0.60^**^	39.380(0.000)
14	3.64 ± 0.92	4.08 ± 0.92^**^	4.75 ± 0.54^**^	42.858(0.000)
15	3.79 ± 0.87	4.10 ± 0.89^**^	4.80 ± 0.47^**^	40.895(0.000)
16	4.17 ± 0.57	4.40 ± 0.73^**^	4.82 ± 0.55^**^	25.291(0.000)
Total score	63.38 ± 9.49	68.40 ± 8.73^**^	75.96 ± 3.52^**^	60.627(0.000)

Comparison of mild and moderate groups marked on moderate group, and that of moderate and severe groups marked on severe group, ^*^P < 0.05;^**^P < 0.01

## Discussion

The Informant Questionnaire is one of the methods used for the clinical assessment of cognitive impairments. Among various questionnaires, the IQCODE is one of the most widely used methods [12]. As the IQCODE is a reliable and validated informant-based instrument for assessing changes in everyday cognitive dysfunction over a 10-year period, it has been widely adopted by clinical researchers across different cultures and languages [8, 24]. In general, informant-based assessments do not rely on direct patient testing, thus making capturing changes over time possible and operable and making these assessments less prone to social-cultural biases [25, 26]. However, having an informant who has known the older individual in question for at least 10 years respond to the questions is a key requirement for the IQCODE [12]. The IQCODE has performed at least as well as cognitive screening tests, such as the MMSE. Furthermore, an important advantage of the IQCODE over the MMSE lies in that it is not affected by the patient’s premorbid intelligence or education [27]. These properties are important for obtaining acceptable diagnostic accuracy, which is required for screening tools [28]. Moreover, from a clinician’s perspective, the strengths of the IQCODE are that it is copyright free, available in different languages, and relatively easy to complete. From the original version with 26 questions that was developed in 1989, the short 16-item IQCODE version was developed in 1994, and its performance was essentially identical to that of the original version [16, 29]. There have been many studies supporting the validity of the IQCODE for dementia screening, with a sensitivity ranging from 79 to 100% and a specificity ranging from 68 to 100% [16, 30, 31]. However, no studies evaluating the efficacy of IQCODE for assessing the severity of cognitive impairments in patients with AD have been published.

This study was based on the 16-item version, and the cognitive changes were scored on a 5-point scale. Higher scores corresponded to greater cognitive decline. The ratings were averaged, composing a mean score between 1 and 5. The severity of cognitive impairments of the total of 394 AD patients was assessed by the CDR and the short IQCODE and was tested by the MMSE, DRS and ADAS-Cog. These patients were classified into mild, moderate and severe dementia groups according to the severity of dementia. Our results demonstrated that in the moderate group, there was a statistically significant association between the IQCODE scores and the MMSE, DRS and ADAS-Cog scores. However, no statistical significance was observed in the mild and severe groups. These findings suggest that IQCODE is more accurate and efficient for assessing moderate dementia than for assessing mild or severe dementia.

Significant difference in each item in the 16-item IQCODE were found in the mild and moderate groups but not in the severe group. ROC curve analyses for the IQCODE discriminating between mild-moderate dementia showed an AUC of 0.666. The sensitivity was 66.1%, and the specificity was 59.8% with 65 used as the cut-off point. The AUC under the ROC curve for the IQCODE discriminating between moderate-severe dementia was 0.768; the sensitivity was 73.9%, and the specificity was 67.7% when using 75 as the cut-off score. Our results revealed that the sensitivity and specificity of the IQCODE were not high enough. Hence, we proposed that there might be a relatively large deviation when using the IQCODE alone to assess the severity of cognitive impairments. Furthermore, the IQCODE is unsatisfactory for evaluating conditions or treatment effects of patients with AD. Collectively, we do not advocate the use of the IQCODE alone for assessing treatment efficacy. A combination of the IQCODE with objective cognitive function tests and evaluation of patients’ behavioural and psychiatric symptoms is required to provide the basis for further treatment.

## Conclusions

In conclusion, with the application of the IQCODE to survey 394 patients, we believe that the IQCODE is moderately effective for assessing the severity of cognitive impairments in AD patients. However, it is recommended that other clinical dementia rating scales be used to supplement the IQCODE in order to increase the sensitivity and the specificity and avoid misjudgements.

## Abbreviations

AD: Alzheimer’s disease
ADAS-Cog: Alzheimer’s Disease Assessment Scale- Cognitive Subscale
AUC: the area under the curve
CDR: Clinical Dementia Rating
DRS: Mattis Dementia Rating Scale
IQCODE: Informant Questionnaire on Cognitive Decline in the Elderly
MMSE: Mini-Mental State Examination
ROC: Receiver operating characteristic
